# Methadone maintenance—lessons from two systems in China

**DOI:** 10.1186/s12954-017-0193-7

**Published:** 2017-09-25

**Authors:** Shui-Shan Lee, Robert Newman

**Affiliations:** 1 Stanley Ho Centre for Emerging Infectious Diseases, The Chinese University of Hong Kong, 2/F Postgraduate Education Centre, Prince of Wales Hospital, Shatin, Hong Kong China; 2 Beth Israel Medical Center (President Emeritus), New York, USA

**Keywords:** Methadone maintenance treatment, MMT, Harm reduction, Drug rehabilitation, HIV, Human immunodeficiency virus

## Abstract

In Hong Kong, methadone maintenance treatment (MMT) was launched in the 1970s, almost 30 years before the counterpart programme’s inauguration in Mainland China. Both were established in response to perceived public crises—addiction-related crime and HIV outbreak, respectively—and both are now regular services under two systems in the same country. Effectiveness of MMT in achieving the stated goals was evident in each case and provides useful lessons on strategies for dealing with the varied concerns related to illicit drug use. Today, with changing patterns of drug addiction, increasing competition for resources, and changing attitudes towards addiction and its treatment, the two MMT systems are confronted with similar challenges to achieve sustainability.

## Background

The introduction of methadone maintenance treatment (MMT) first emerged more than half a century ago amidst growing concern over the opiate addiction problem [1]. Subsequently, in the 1980s, the escalating incidence of human immunodeficiency virus (HIV) infection in people who inject drugs (PWID) led to the positioning of MMT as part of an intervention now widely known under the rubric ‘harm reduction’ [2]. In essence, harm reduction acknowledges the reality that, as of today, addiction is not curable but can be and should be treated.

Addiction, related criminology and HIV transmission are related public health and security threats that have put the performance of MMT to the test. In Hong Kong, currently a special administrative region of China and in the 1970s a colony of United Kingdom, MMT was set up as a response to concern over increasing crime related to the illicit use of and trafficking in opiates and in Mainland China as an effort to curb the spread of HIV [3]. The history of MMT in these two regions, marked by very different political, social, and economic characteristics, is in some respects similar, and in others quite distinct. Experiences from the two programmes offer valuable lessons to authorities within and outside China.

## Programmes driven by crises

Sadly, public health interventions are often initiated only when a threat has escalated to ‘crisis’ level. For infections, outbreaks are the visible signal of crisis in the eyes of the government. While the spread of heroin addiction does in some ways mimic infectious disease outbreaks, the public’s health was not the primary concern of the Hong Kong government in the 1970s. At that time, Hong Kong had a developing economy threatened with a high crime rate that to a large extent was attributed to heroin abuse. It was also the time when news of potentially effective outcomes of MMT reached the local medical community [1], leading to the launching of a small-scale pilot methadone treatment clinic. In 1975, however, the government of Hong Kong engaged an overseas consultant to examine the situation and recommend a response that might be capable of a prompt, affordable and effective way to proceed on the scale necessary. The outcome was development of what rapidly developed into a methadone-based treatment programme that could offer prompt treatment to every opiate-dependent person in Hong Kong who wanted and needed it. Within just 2 years, the programme, comprising over 20 clinics, reached an average attendance of some 10,000 drug users daily [4].

Across the border, on the Mainland, methadone treatment was eschewed for decades, and heroin addiction continued to be confronted by the same punitive policy that had been the hallmark of the People’s Republic of China since its establishment. In the early 1990s, however, the Chinese government was suddenly faced with an alarming rise in HIV prevalence—first in Yunnan [5] and then in other provinces, notably Guangxi and Xinjiang [6, 7]. The concerns heightened when the HIV epidemic appeared to be affecting other populations, e.g., female sex workers [8]. While awaiting policy directives from the government, local health professionals were keen to start MMT. The wait, however, proved a long one, but ultimately, eight pilot clinics were approved and were set up in 2004 [9]. The pilot phase quickly grew into the establishment, by 2006, of 128 clinics.

## Pathways to distinctive service models

In Hong Kong, there had been only one government-run pilot programme prior to full implementation of territory-wide MMT services. While motivated primarily by the perceived need to enhance public security rather than as a public health initiative, MMT was, of course, a medical service, whose effectiveness was dependent on its appeal to participants and on its ability to assist in significantly reducing the self-administration of opiates. Heroin users were encouraged to register at any convenient MMT clinic and pay $HK1 (then and now approximately 12 cents in US currency) for a daily dose of methadone, the same fee charged in the 1970s by all government-operated health facilities. Today, the MMT per visit fee for Hong Kong residents remains $HK1, while the charge for attendees of general outpatient clinics has risen 45-fold!

With the support of a handful of medical doctors managing the entire programme, most of them on a part-time basis, frontline services (including preparing and administering methadone) have from the outset been shouldered by trained non-medical personnel, recruited from the Hong Kong ‘Auxiliary Medical Service’ and paid on an hourly basis [4]. The low threshold, hassle-free approach has continued to this day, with a registered programme caseload of approximately 10,000. It has been estimated that over 60% of heroin users in Hong Kong attend the clinics daily [4], and a study of a randomly selected group of heroin users found that over 90% were receiving methadone [10].

**Fig. 1:**
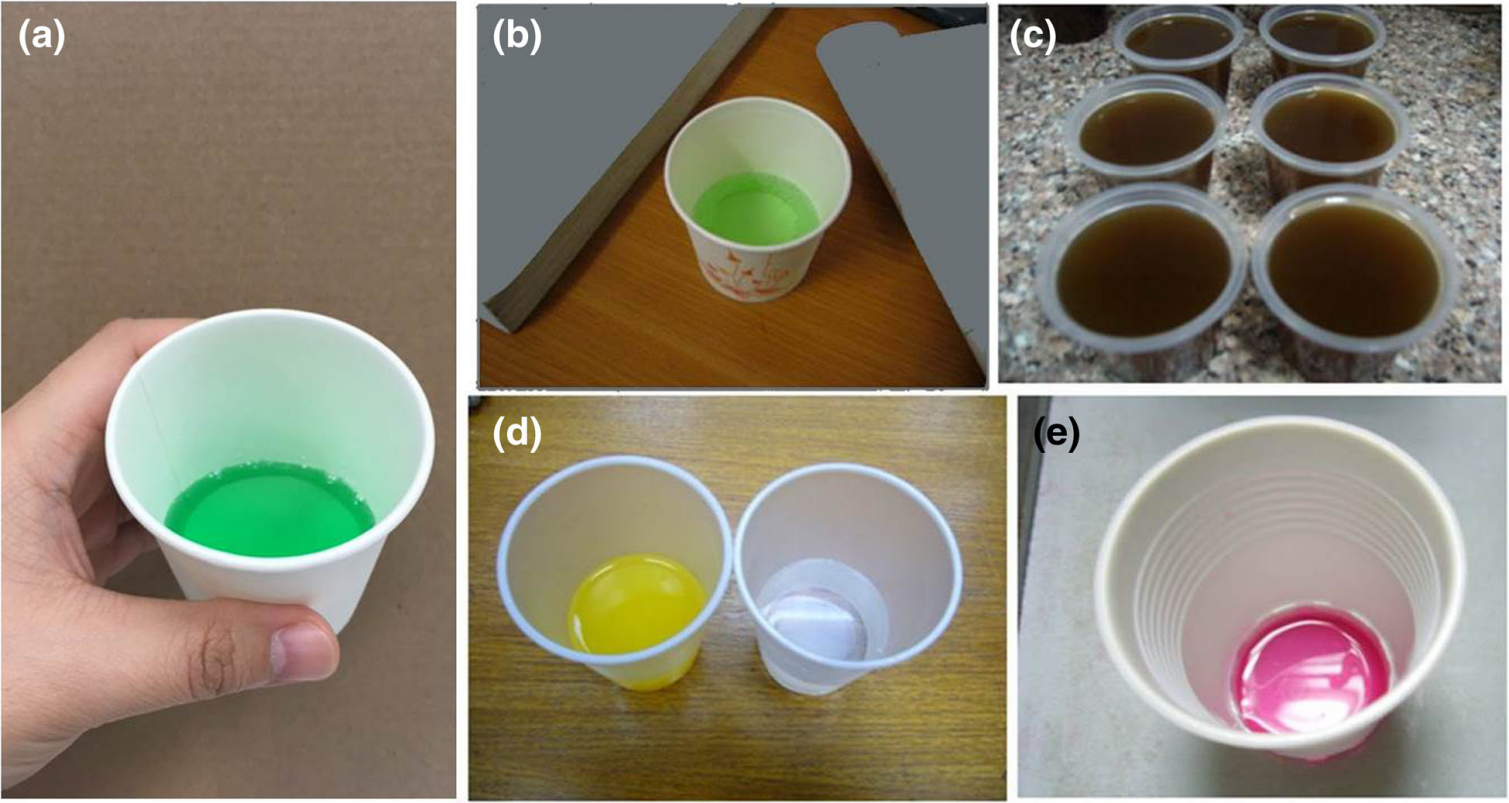
Methadone (**a**) is handed over to drug users under direct supervision of staff at Hong Kong’s methadone clinics. In the years before and following the millennium, professionals in neighbouring cities made study tours to Hong Kong to take reference of the latter’s methadone maintenance treatment (MMT) service. The model of care varied, alongside the colour of methadone: **b** green in cities in Mainland China, for example, Liuzhou; **c** coffee-like in Kuala Lumpur, Malaysia; **d** colourless in Macau, offered in conjunction with orange-coloured laxative; and **e** pink in Taiwan

Between the mid 1990s and 2004, Hong Kong was considered a potential model for cities in the Mainland and South East Asia in their planning of treatment services that might achieve the goal of harm reduction (Fig. 1). Many visitors were impressed by the voluntary attendance of so many patients at the very modestly staffed and financed clinics. And yet, this pragmatic, relatively inexpensive, rapidly expandable treatment model was adopted only rarely. Among the major criticisms: the treatment was ‘too simple’ and unsophisticated, and lacked the comprehensive array of ‘supportive services’ that in many countries of the world were (and continue to be) mandated by regulation and that patients are required to accept as a condition of receiving medication.

In Mainland China, development of MMT took a different path. The primary focus has been on reducing the incidence of HIV among PWID. Initially, stringent admission criteria were applied, but these have subsequently been relaxed. For example, PWID without *houkou* (proof of registration in their home town) were previously ineligible to receive treatment in any other locale; that restriction has since been eliminated. The clinics are staffed by doctors and ancillary support personnel and are permitted to levy up to RMB10 (~ $US1.2) per visit [11]. Despite a fee that is some tenfold greater than that of Hong Kong, as of the end of 2011, over 700 clinics were in operation with over 340,000 voluntary attendees recorded in the database [12]. Against the background of over one million registered drug users [13], however, the coverage remains quite low.

As the world’s largest MMT programme, many research reports have appeared from China in the past decade. For example, a major fall of the HIV incidence rate, from a two-digit to a one-digit percentage, has been reported [11, 14], though the extent was variable [15]. Apart from reduction of risk behaviours [11], a notable decrease in drug-related crimes has been observed [16].

In Hong Kong, paradoxically, very little research has been conducted throughout the 40 years of MMT service, and this has been a shortcoming in the eyes of local as well as overseas critics. The limited published studies have focused on reduction of behavioural risks in methadone users [17], but since the HIV rate has from the outset been extremely low, no quantifiable impact on spread has been measurable. However, it is certainly noteworthy that the very sharp rise in HIV incidence in neighbouring countries and in other nations around the world has not been experienced, and the consistently low number of new cases among PWID in Hong Kong has been attributed to the functioning of MMT [4]. On the other hand, there has been documentation of the reduction in crime associated with illicit drug use [18].

## The challenge of maintaining methadone-based treatment services

In Hong Kong, the continued decrease in the prevalence of heroin use (http://www.nd.gov.hk/en/crda_report.htm) has been associated with a steady fall in MMT caseload [19]. The daily attendance was over 10,000 in the 1970s and 1980s, falling to 8000 in 1990, 6502 in 2000 and 5702 in 2013 (Department of Health Annual Reports http://www.dh.gov.hk/) [20]. This experience, along with the persistently very low HIV prevalence in PWID, appears to have lowered the priority given to MMT by local authorities as well as by the public at large and has been accompanied by increasing criticism of the programme’s goals and operations. For example, in 2008, the Hong Kong government’s Audit Commission criticised the methadone clinics for failing to rid heroin users of the addiction habit and for the decreased cost-effectiveness related to lower caseloads cared for by the same number of clinics (Director of Audit’s report no. 50 http://www.aud.gov.hk/) [21].

Regarding the criticism that the MMT in Hong Kong has failed to pursue ‘completion of treatment’ as a therapeutic objective, this simply reflects the programme’s acceptance of the undeniable fact that, to date, it is not known how to ‘cure’ opiate addiction and that it is therefore important to assist patients to achieve abstinence from illicit opiates ‘1 day at a time’. This, of course, is a concept that for many decades has been the fundamental orientation of Alcoholics Anonymous, and in that setting, it has been almost universally accepted. When adopted by providers of opiate dependency treatment, however, it is widely rejected, and that seems increasingly to be the attitude in Hong Kong.

Across the border in Mainland China, the MMT retention rate has been quite low from the outset [22], and this might be expected to threaten the continued enthusiasm of policymakers, especially if the HIV prevalence continues to drop. Ever since the introduction of harem reduction prgrammes, HIV prevalence in Mainland China had fallen from a peak of 30.3% in 1999 to 10.9% in 2011 [23]. If indeed there is a significant cooling of the Chinese government’s continued willingness to support MMT to the same extent as heretofore, it might be associated with a renewed emphasis on law enforcement as the primary approach to addiction, notwithstanding its acknowledged lack of success in the past.

The cost of MMT is another concern, both in Hong Kong and on the Mainland. In Hong Kong, the cost has been kept low by virtue of a very small professional staff-base and a limited array of services within the clinics. The result is a programme that has to date been deemed affordable both by government and the patients who are charged the nominal fee of $HK1 per visit. And yet, as noted above, even these moderate costs were criticised by the Hong Kong government’s Audit Commission [21]. Interestingly, some Mainland residents with eligible travel documents do shuttle to Hong Kong for daily doses of methadone that are available without an array of demands imposed by programme rules and regulations. Of course, the relatively high per diem out-of-pocket cost to patients in China relative to that in Hong Kong also is a factor.

Clearly, governmental support is a sine qua non for launching and continuing methadone-based treatment of opiate dependence, and government support in turn depends in large measure on the community’s attitude. In Hong Kong, location of clinics and social connectivity of methadone users were important in ensuring high levels of acceptance of and adherence to MMT on the part of the target population [24]. Attempts to adopt a business model through such means as consolidation of small clinics with the goal of achieving higher cost-effectiveness are surely tempting but must be pursued with great caution as this may compromise the ability of the programme to attract and retain those who most need the help that is being offered.

It is clearly essential to maintain the awareness of the general public of the key role that treatment services, and methadone-based treatment in particular, can and must play to serve those who are afflicted as well as the society in general. A very specific example of opposition that exists in Hong Kong, Mainland China and many other countries affected by opiate addiction is the not-in-my-backyard (NIMBY) syndrome. The potential for rejection by local communities of MMT clinic operating in their midst is a threat that can and must be anticipated and, hopefully, thwarted through constant education.

## Conclusion

Despite the varied social environments and circumstances surrounding the launch and expansion of MMT in Hong Kong and Mainland China, both have demonstrated the ability to respond rapidly and effectively if the commitment is there to do so. This is the key lesson learned from two seemingly different programmes in varied setting. However, it is essential that the challenge of sustainability be recognised and addressed. The changing HIV epidemiology, addiction habits, competition for resources and the community’s attitude are factors that cannot be ignored if the critically important treatment services are to be sustained and, as needed, to be expanded.

Perhaps the most important lessons of the experience both in Hong Kong and on the Mainland are that significant numbers of opiate-dependent individuals *want* help, *need* help and *respond well* when treatment is made available and that *the goal of ensuring prompt availability, affordability and acceptability of treatment services for opiate dependence is achievable. The one and only essential factor needed to succeed is, again, commitment.*

## Abbreviations

HIV: Human immunodeficiency virus
MMT: Methadone maintenance treatment
NIMBY: Not-in-my-backyard
PWID: People who inject drug
